# IL-17 Receptor Signaling Negatively Regulates the Development of Tubulointerstitial Fibrosis in the Kidney

**DOI:** 10.1155/2018/5103672

**Published:** 2018-10-14

**Authors:** Kritika Ramani, Roderick J. Tan, Dong Zhou, Bianca M. Coleman, Chetan V. Jawale, Youhua Liu, Partha S. Biswas

**Affiliations:** ^1^Division of Rheumatology and Clinical Immunology, Department of Medicine, University of Pittsburgh, Pittsburgh, PA 15261, USA; ^2^Renal-Electrolyte Division, Department of Medicine, University of Pittsburgh, Pittsburgh, PA 15261, USA; ^3^Department of Pathology, University of Pittsburgh, Pittsburgh, PA 15261, USA

## Abstract

Chronic inflammation has an important role in the development and progression of most fibrotic diseases, for which no effective treatments exist. Tubulointerstitial fibrosis (TF) is characterized by irreversible deposition of fibrous tissue in chronic kidney diseases. Prolonged injurious stimuli and chronic inflammation regulate downstream events that lead to TF. In recent years, interleukin-17 (IL-17) has been strongly linked to organ fibrosis. However, the role of IL-17 receptor signaling in TF is an active area of debate. Using the unilateral ureteral obstruction (UUO) mouse model of TF, we show that IL-17 receptor A-deficient mice (*Il17ra^−/−^*) exhibit increased TF in the obstructed kidney. Consequently, overexpression of IL-17 restored protection in mice with UUO. Reduced renal expression of matrix-degrading enzymes results in failure to degrade ECM proteins, thus contributing to the exaggerated TF phenotype in *Il17ra*^−/−^ mice. We demonstrate that the antifibrotic kallikrein-kinin system (KKS) is activated in the obstructed kidney in an IL-17-dependent manner. Accordingly, *Il17ra^−/−^* mice receiving bradykinin, the major end-product of KKS activation, prevents TF development by upregulating the expression of matrix-degrading enzymes. Finally, we show that treatment with specific agonists for bradykinin receptor 1 or 2 confers renal protection against TF. Overall, our results highlight an intriguing link between IL-17 and activation of KKS in protection against TF, the common final outcome of chronic kidney conditions leading to devastating end-stage renal diseases.

## 1. Introduction

Fibrosis affects all vital organs and is responsible for a staggering 45% of deaths in the US [1]. Unresolved inflammation triggers downstream signaling events that lead to organ fibrosis [2, 3]. No better example of this grave clinical scenario exists than in renal tubulointerstitial fibrosis (TF) [4]. TF is characterized by irreversible deposition of fibrous tissue in the tubular space in patients with chronic kidney injury leading to end-stage renal diseases (ESRD) [5]. Every year, approximately half a million ESRD patients receive dialysis or transplantation (http://www.niddk.gov). Inadequate early diagnostic tools and lack of antifibrotic medications further aggravate the prognosis in these patients. Thus, the need for safe and effective drugs to prevent TF is desperately needed in the clinic. However, the development of antifibrotic therapeutic strategies is hindered by our lack of understanding of the profibrotic events in the kidney during chronic kidney diseases.

Renal inflammation confers an initial protective response to injury. However, unresolved inflammation can drive excessive deposition of extracellular matrix (ECM) protein in the tubular space [3, 5]. The profibrotic cascade from initial damage to fibrosis is regulated by cytokines that drive activation of renal tubular epithelial cells and myofibroblasts, macrophage infiltration, and production of TGF*β*, kidney proteases, and growth factors. To date, the primary focus of the renal fibrosis field centers around understanding the origin of myofibroblasts and tissue remodeling processes. Far less effort has been dedicated to dissecting the role of cytokines in regulating profibrotic events. Yet, cytokines remain an appealing therapeutic opportunity, given the clinical success of anticytokine biologics therapy. Few studies have implicated IL-4, IL-5, and IL-13 (Th2 cytokines) in fibrosis [6]. In contrast, under inflammatory settings where IFN-*γ* (Th1 cytokine) dominates, fibrosis is attenuated [6]. Thus, tight regulation of the balance between pro- and antifibrotic cytokines dictates the outcome of profibrotic events, a process that is poorly understood.

The homeostasis of ECM proteins is a highly regulated process, involving multiple proteases and their regulators. An uneven deposition of ECM components either due to increased synthesis or diminished degradation of ECM proteins eventually replaces the normal renal parenchyma with fibrous tissue [7]. Two protease systems, the plasminogen activation system and the matrix metalloproteinase (MMP) family, play a critical role in degrading ECM components in the fibrotic kidney [8]. Each of these proteolytic systems is regulated by their respective endogenous activators and inhibitors, confirming a delicate balance of the activity of these enzymes by both positive and negative regulation.

IL-17 family cytokines (IL17A-F) are produced by T-helper 17 (Th17) cells and other innate IL-17 producers, such as invariant NKT, *γδ* T, natural Th17, and group III innate lymphoid cells [9, 10]. IL-17A (IL-17) bind to cognate receptors (IL-17RA/RC) of target cells and activate Act1, NF-*κ*B, and C/EBP*γ*/*δ* and drive the expression of IL-17-responsive genes [10]. Numerous studies, including work from our lab, demonstrated a critical role for IL-17R signaling in renal inflammation following kidney injury [11–13]. In these settings, IL-17 induces the expression of cytokines and chemokines that facilitate the influx of innate effector cells in the kidney [11]. However, the role of IL-17 in TF development is highly debatable. Consistent with its proinflammatory function, one study has implicated IL-17 as a profibrotic cytokine in TF [14]. IL-17 produced by kidney infiltrating *γδ*^+^ T and CD4^+^ T cells were shown to mediate fibrosis via RANTES-driven influx of inflammatory cells. Contrary to this study, a recent report demonstrated a surprising antifibrotic role of IL-17 in the TF following ureteral obstruction [15]. Likewise, IL-17 prevents TF development in deoxycorticosterone acetate + angiotensin II-induced fibrosis in the kidney [16]. Nevertheless, the contribution of IL-17R signaling in the progression of TF is poorly understood.

Our published study showed that IL-17 activates the kallikrein-kinin system (KKS) through the induction of renal expression of kallikrein 1 (Klk1) during systemic fungal infection [17]. Klk1 is a serine protease that cleaves high and low molecular weight kininogens to generate kinins and bradykinin [18]. Bradykinin via bradykinin receptor b1 (Bdkrb1) and bradykinin receptor b2 (Bdkrb2) regulates blood pressure. Additionally, bradykinin receptor activation plays a key role in renal protection [19]. Mice lacking components of the KKS or humans with polymorphisms in KKS-related genes exhibit increased risks for chronic renal disorders [19–24]. Interestingly, mice deficient in Bdkrb1 or Bdkrb2 also show exaggerated TF following kidney injury [25, 26]. In this setting, Bdkrb1 or Bdkrb2 activation induces the expression of matrix-degrading enzymes that facilitate the removal of ECM proteins and favor healing in the injured kidney. Although activation of KKS is linked to many acute and chronic kidney diseases, regulation of KKS in the kidney particularly during TF remains remarkably understudied in comparison to other organs including skin, salivary glands, and liver.

Here, we show that IL-17 is upregulated and plays an unappreciated antifibrotic role in the kidney following UUO. Accordingly, mice deficient in *Il17ra* signaling show exaggerated fibrosis in the obstructed kidney and overexpression of IL-17 prevented TF development in WT mice. Failure to degrade ECM proteins due to reduced renal expression of matrix metalloproteinase-2 (MMP2) and tissue plasminogen activator (tPA) contribute to the exaggerated TF phenotype in *Il17ra^−/−^* mice. Mice lacking the IL-17 receptor A subunit (*Il17ra^−/−^*) exhibited diminished Klk1 expression in the kidney. Treatment with bradykinin reduced TF development in *Il17ra^−/−^* mice via upregulating Mmp2 and tPA levels in the obstructed kidney. Finally, we show that both Bdkrb1 and Bdkrb2 are equally required for protection against TF. These data identify a previously unrecognized link between IL-17 and KKS-mediated renal protection against TF, which may provide the basis for clinical intervention in this disease.

## 2. Materials and Methods

### 2.1. Mice

C57BL/6 (WT) mice were obtained from Jackson Laboratory (Bar Harbor, ME). IL-17 receptor A-deficient (*Il17ra*^−/−^) mice on the C57BL/6 background were generously provided by Amgen (San Francisco, CA) and bred in-house. 8- to 10-week-old male mice were used for all the experiments. All studies were carried out under approved protocols of the University of Pittsburgh Institutional Animal Care and Use Committee adhering to the guidelines in the Guide for the Care and Use of Laboratory Animals of the National Institutes of Health.

### 2.2. Unilateral Ureteral Obstruction Model of Kidney Fibrosis

Unilateral ureteral obstruction (UUO) was executed as described previously [27]. Briefly, under anesthesia, the left ureter was isolated and ligated 2–4 mm below its origin (UUO kidney). The right ureter is left unobstructed (non-UUO kidney). Seven days post-surgery, mice were sacrificed, and the UUO and contralateral non-UUO kidneys were harvested for further analysis.

### 2.3. Morphological and Histological Analysis for UUO

The UUO and non-UUO kidneys were fixed in formalin, dehydrated, and paraffin-embedded. Serial sections (4–5 *μ*m) were stained with periodic acid-Schiff (PAS) (Sigma-Aldrich, St. Louis, MO). To visualize kidney fibrosis via detection of total collagen, serial kidney sections were stained with Masson's trichrome and Picrosirius red stains, as described before (Sigma-Aldrich, St. Louis, MO). Morphometric analysis of the tubular inflammation and interstitial fibrosis was evaluated as previously described [28].

### 2.4. Immunofluorescence Staining

Frozen sections (5 *μ*m thickness) were fixed in acetone and blocked with 1% BSA in PBS. The staining for collagen I, collagen III, and *α*SMA was performed using FITC-conjugated mouse anti-collagen I, anti-*α*SMA (SouthernBiotech, Birmingham, AL), and anti-collagen III antibodies (Novus Biologicals, Littleton, CO), respectively. Slides were mounted with Vectashield mounting medium with or without 4′,6′-diamidino-2-phenylindole (Vector Labs, Burlingame, CA) and visualized using an EVOS FL Auto microscope (Life Technologies, Carlsbad, CA).

### 2.5. Hydroxyproline Assay

Hydroxyproline is a component largely seen only in collagen. Measurement of hydroxyproline levels can therefore be used as an indicator of collagen content. 10 mg of kidney tissue was homogenized with 12 M HCl (Sigma-Aldrich, St. Louis, MO) at 120°C overnight. Hydroxyproline levels were quantified using a commercially available hydroxyproline assay kit (Chondrex, Redmond, WA) following the manufacturer's instructions. The samples were finally read at 560 nm. Values were represented as mg collagen/mg kidney tissue.

### 2.6. Isolation of RNA and Real-Time PCR

Total RNA was isolated from kidney tissue by using the RNeasy Micro Kit according to the manufacturer's instructions (Qiagen, Valencia, CA). cDNA was synthesized using SuperScript III First-Strand (Invitrogen, Carlsbad, CA). Gene expression was measured by qPCR with PerfeCTa SYBR Green FastMix ROX (Quanta BioSciences, Gaithersburg, MD) on the 7300 Real-Time PCR System (Applied Biosystems, Carlsbad, CA). Gene expression levels were then determined for target genes using commercially available QuantiTect primers (Qiagen, Valencia, CA) by real-time PCR. The expression of each gene was normalized to that of GAPDH.

### 2.7. Flow Cytometry Analysis of Single-Cell Suspension from the Kidney

Kidneys were perfused with 15 mL prewarmed PBS. The perfused kidneys were digested with collagenase B (0.230 U/mL; Roche Applied Science, Indianapolis, IN) in 10% FCS in RPMI for 30 min (37°C) with occasional shaking. RBC lysis was performed, and cells were resuspended in 10 mL RPMI media. The cells were slowly layered over 5 mL Lympholyte-M (Cedarlane, Burlington, Canada). The tubes were spun for 1200 rpm for 30 min at room temperature. The cell layer at the interface of the media and Lympholyte-M was collected and washed twice with PBS and used for flow cytometry staining. The single-cell suspensions from the kidney were surface-stained with fluorochrome-conjugated antibodies against Ly6G (clone: IA8; eBioscience, San Diego, CA, USA), Ly6C (clone: AL-21; eBioscience, San Diego, CA, USA), CD11b (clone: M1/70; eBioscience, San Diego, CA, USA), and F4/80 (clone: BM8; eBioscience, San Diego, CA, USA) followed by flow cytometry. The data were analyzed using FlowJo software (Tree Star, Ashland, OR).

### 2.8. Adenoviruses

Adenoviruses expressing IL-17A (Ad-IL-17) and control (Ad-ctrl) were kindly provided by Dr. J. Kolls (U. Pittsburgh). Mice were injected via the tail vein with 1 × 10^9^ pfu of Ad-IL-17 or Ad-ctrl virus 72 hours prior to UUO surgery.

### 2.9. Oral Antibiotic Treatment

Mice were provided *ad libitum* autoclaved drinking water supplemented with ampicillin (0.5 mg/mL, Sigma-Aldrich, St. Louis, MO), metronidazole (0.5 mg/mL Sigma-Aldrich, St. Louis, MO), neomycin (0.5 mg/mL, Med-Pharmex, Pomona, CA), vancomycin (0.25 mg/mL, Nova Plus, New York, NY), and sucralose (4 mg/mL, Splenda, McNeil Nutritionals, LLC). Splenda was added to make the antibiotic cocktail more palatable. Control mice received autoclaved water with Splenda only. Antibiotic treatment was started 2 weeks prior to UUO surgery and continued for the duration of the experiment.

### 2.10. Bradykinin and Bradykinin Receptor Agonists

Mice were injected i.p. with 200 *μ*L bradykinin (300 nmol/kg/day) (R&D Systems, Minneapolis, MN). Control mice received equal volumes of PBS. For experiments with selective agonists, mice received i.p. injection of Bdkrb1 (1 mg/kg/day) or Bdkrb2 (750 nmol/kg/day) selective agonists as indicated (R&D Systems, Minneapolis. MN). Control mice received equal volume of PBS.

### 2.11. Western Blots

Kidney tissues (10 mg) were homogenized in RIPA buffer (Thermo Scientific, Pittsburgh PA). Concentration of protein was quantitated by the BCA quantitation assay (Thermo Scientific, Pittsburgh, PA). Equal amounts of sample were subjected to electrophoresis and transferred to PVDF membranes (Millipore, Billerica, MA). After blocking with 5% milk in TBS, the blots were incubated with anti-mouse Klk1 (LifeSpan Biosciences, Seattle, WA) or anti-mouse beta-actin (Abcam, Cambridge, MA) overnight in 4°C. The blots were then washed and incubated for 1 hour at room temperature with individual secondary antibodies accordingly. Bands were detected using an enhanced chemiluminescence detection system (Thermo Scientific, Pittsburgh, PA) and developed with a FluorChem E imager (ProteinSimple, San Jose, CA).

### 2.12. Gelatin Zymography

Gelatin zymographic analysis of MMP2 and MMP9 proteolytic activity in kidney tissue homogenates was performed according to the method described previously [27]. Briefly, kidney homogenates were prepared essentially according to the methods described before [29]. Protein concentration was determined using a bicinchoninic acid (BCA) protein assay kit. A constant amount of protein (30 *μ*g) from the kidney tissue homogenates was loaded onto commercially 10% Zymogram (gelatin) gel (Life Technologies, Carlsbad, CA). After electrophoresis, proteinase activity was detected as unstained bands on a blue background representing areas of gelatin digestion, as per the manufacturer's protocol.

### 2.13. Statistical Analyses

Results are expressed as mean ± SD. Differences between groups were calculated for statistical significance using 2-tailed paired Student's *t* tests and ANOVA as appropriate. A *p* value less than 0.05 was considered significant.

## 3. Results

### 3.1. IL-17 and IL-17-Responsive Genes Are Upregulated in the Obstructed Kidney

**Figure 1:**
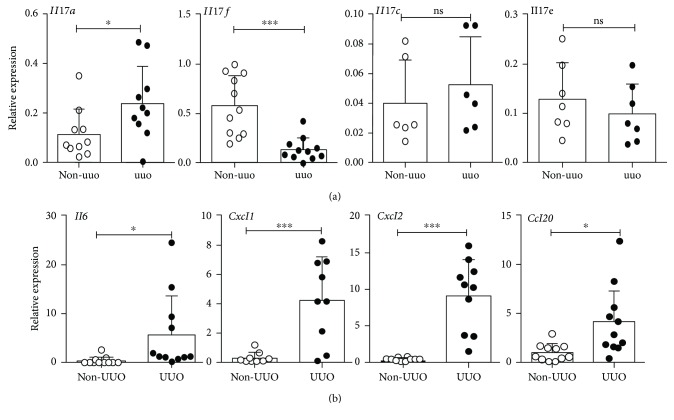
IL-17 and IL-17 target gene expressions are increased in the obstructed kidney of WT mice. WT mice were subjected to UUO (*n* = 7–11). Mice were sacrificed at day 7 post-surgery, and renal transcript expressions of (a) Il17a, Il17f, Il17c, and Il17e, and (b) IL-17-responsive genes (Il6, Ccl20, Cxcl1, and Cxcl2) in the UUO and non-UUO kidneys were evaluated by qPCR. In the scattered plots, each dot represents individual mouse and error bars indicate mean ± SD. The data are pooled from three independent experiments. *p* value ≤0.05 (^∗^), ≤0.001 (^∗∗∗^), and ≤0.0001 (^∗∗∗∗^). ns: not significant.

To determine the expression of IL-17 family of cytokines in the obstructed kidney, WT mice were subjected to UUO and renal transcript expression of *Il17a*, *Il17f*, *Il17c*, and *Il17e* were evaluated by qPCR at day 7 post-surgery. Confirming previous reports, we observed a significant increase in *Il17a* mRNA expression in the UUO kidney compared to non-UUO (Figure 1(a)) [14]. While the *Il17f* mRNA level was reduced in the obstructed kidney, transcript expressions of *Il17c* and *Il17e* were comparable between the UUO and non-UUO kidneys.

We next assessed the functional consequence of IL-17A (IL-17) production in the UUO kidney by measuring the transcript expression of IL-17-responsive cytokines and chemokine genes. The mRNA levels of *Il6*, *Ccl20*, *Cxcl1*, and *Cxcl2* were significantly elevated in the UUO kidney compared to non-UUO at day 7 post-UUO surgery (Figure 1(b)). These results indicate that expressions of IL-17A (IL-17) and IL-17-responsive inflammatory genes are increased in the kidney following ureteral obstruction.

### 3.2. IL-17RA Signaling Is Critical for Protection against TF following UUO

**Figure 2:**
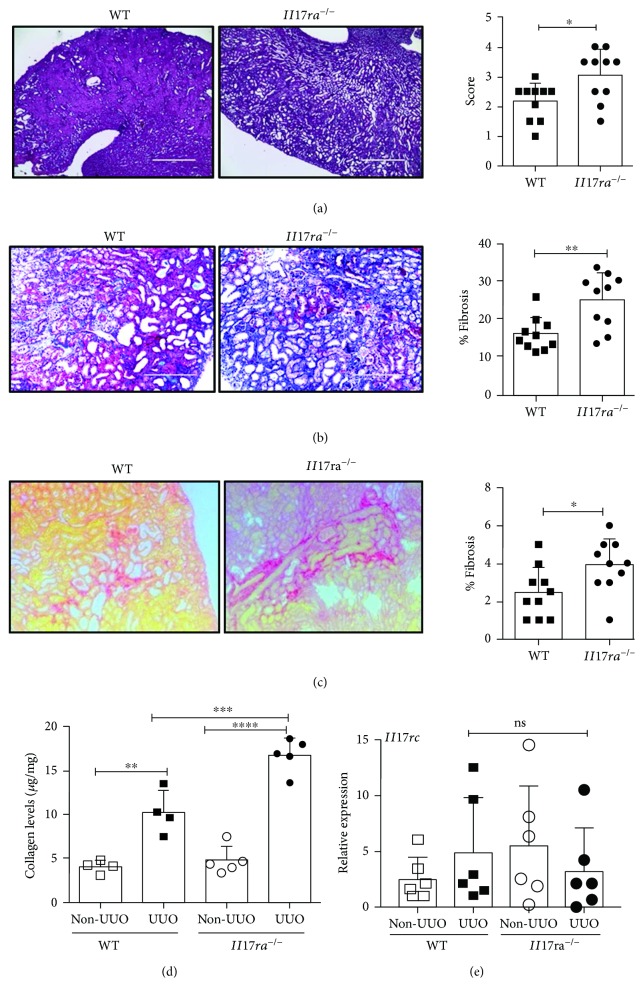
Exaggerated TF in Il17ra^−/−^ mice following UUO. WT and Il17ra^−/−^ mice (*n* = 10) were subjected to ureteral obstruction. At day 7 post-surgery, UUO kidneys were stained with (a) PAS, (b) Masson's trichrome, and (c) Picrosirius red stain to evaluate inflammatory changes and TF development. The inflammation score and percentage fibrosis were quantified blindly based on stained sections. Photomicrographs are representative of 2 independent experiments. Original magnification: 4x (a), 20x (b), and 10x (c). (e) The absolute collagen content in the UUO and non-UUO kidneys (*n* = 4–5) was quantified using hydroxyproline assay. (e) Kidneys were evaluated for Il17rc mRNA expression by qPCR. In the scattered plots, each dot represents individual mouse and error bars indicate mean ± SD. The data are pooled from two independent experiments. *p* value ≤0.05 (^∗^), ≤0.01 (^∗∗^), ≤0.001 (^∗∗∗^), and ≤0.0001 (^∗∗∗∗^).

To define the contribution of IL-17RA signaling in TF, WT and *Il17ra^−/−^* mice were evaluated for renal tissue damage and fibrosis at day 7 post-UUO surgery. Surprisingly, mice deficient in *Il17ra* signaling demonstrated significantly increased tissue damage and collagen deposition in the renal parenchyma, as evidenced by PAS (Figure 2(a)), Masson's trichome (Figure 2(b)), and Picrosirius red staining, respectively (Figure 2(c)). The non-UUO kidneys from WT and *Il17ra^−/−^* mice showed no fibrotic changes. In line with the histopathology data, we observed a significant increase in the total collagen content in the obstructed kidneys of *Il17ra^−/−^* mice than WT animals (Figure 2(d)). Moreover, to ensure that the observed phenotype in *Il17ra*^−/−^ mice is not due to increased IL-17 signaling via IL-17RC, we measured *Il17rc* mRNA level in the obstructed and nonobstructed kidneys of WT and *Il17ra*^−/−^ mice. As shown in Figure 2(e), *Il17rc* transcript level was comparable between the groups. These results highlight an unexpected antifibrotic role for IL-17RA signaling in the pathogenesis of TF.

**Figure 3:**
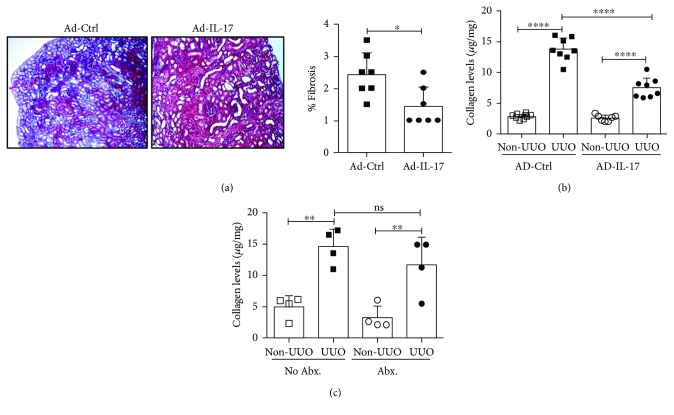
Overexpression of IL-17 reduced TF development in the WT mice. WT mice (*n* = 7–8) were either injected with adenovirus overexpressing IL-17 (Ad-IL-17) or control vector (Ad-ctrl) (10^9^ pfu) 72 h prior to performing UUO surgery. The TF development was evaluated at day 7 post-surgery by (a) Masson's trichrome staining of serial sections from UUO kidneys. Photomicrographs are representative of 2 independent experiments. Original magnification: 10x. (b) The total collagen content in the kidneys was evaluated by hydroxyproline assay. (c) WT mice (*n* = 4) were treated with a cocktail of antibiotics (Abx) in drinking water starting day 7 (relative to UUO surgery) and then throughout the experiment. Control mice (no Abx treatment) received drinking water only. At day 7 post-surgery, total collagen content in the kidney was evaluated by hydroxyproline assay. In the scattered plots, each dot represents individual mouse and error bars indicate mean ± SD. The data are pooled from two independent experiments. *p* value ≤0.05 (^∗^), ≤0.01 (^∗∗^), and ≤0.0001 (^∗∗∗∗^). ns: not significant.

Since IL-17RA serves as a receptor subunit for *Il17*, *Il17f*, *Il17c*, and *Il17e*, we next sought to verify the renal protective role for IL-17 in TF [10]. To that end, we infected WT mice either with an adenovirus overexpressing IL-17 (Ad-IL-17) or adeno-control vector (Ad-Ctrl). We have previously shown that Ad-IL-17 infection in mice resulted in 200-fold more serum IL-17 than the Ad-ctrl vector, which could be detected 6 days post-Ad-IL-17 injection (last time point analyzed) [17]. Additionally, the increase in IL-17 level was not associated with systemic inflammation, as serum TNF*α* and IL-1*β* levels were undetectable [17]. In comparison to mice injected with Ad-ctrl, IL-17 overexpression led to significantly reduced fibrotic changes and total collagen deposition in the obstructed kidney at 7 days post-UUO (Figures 3(a) and 3(b)). Overall, our results suggest that IL-17 prevents the deposition of collagen in the tubulointerstitial space following UUO.

The role for IL-17 in the TF development has been conflicting based on existing literature. A previous study by Peng et al. identified IL-17 as a profibrotic cytokine in the mouse model of UUO [14]. In stark contrast, a recent study has shown increased TF in the absence of IL-17 following ureteral obstruction [15]. The apparent discordance between these findings is presently unclear. It is possible that difference in the gut microbiome, shown to drive IL-17 response in the kidney diseases, may account for the seeming disagreement between the study by Peng et al. and our results [30]. To define the involvement of the gut microbiome in IL-17-driven TF, we depleted gut microbiota by treating WT mice with a cocktail of antibiotics before performing UUO surgery. Mice treated with antibiotics showed comparable level of total collagen content in the obstructed kidney than did untreated mice (Figure 3(d)). These results suggest that differences in the gut microbiome between mice may not account for the disagreement between the TF phenotypes observed by Peng et al. and our studies.

### 3.3. Increased Synthesis of ECM Proteins Does Not Contribute to the Exaggerated TF in *Il17ra^−/−^* Mice

**Figure 4:**
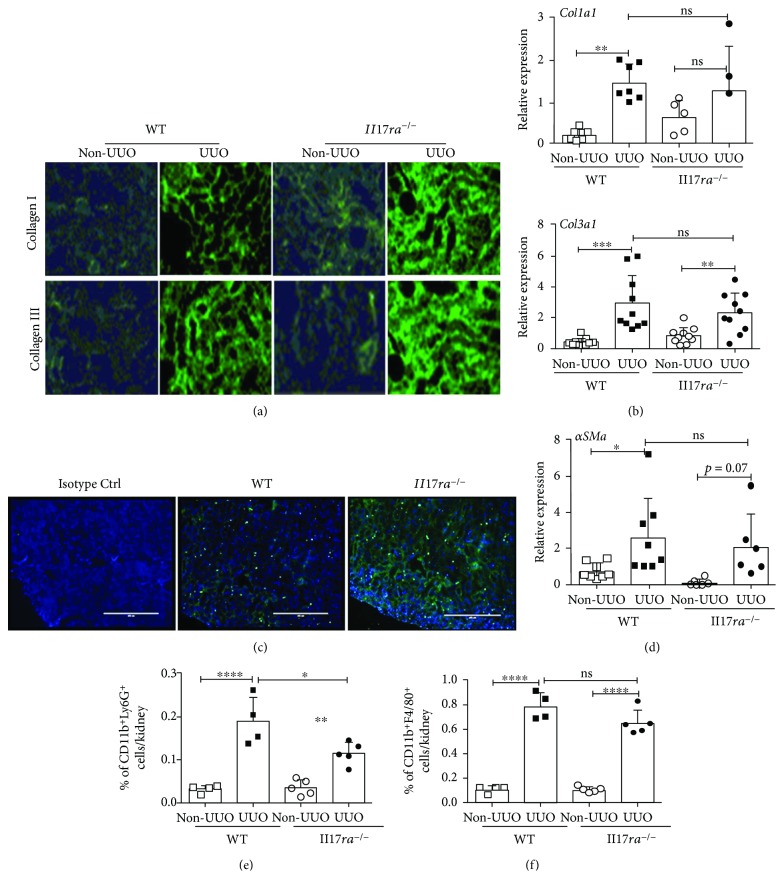
Diminished ECM protein degradation and not synthesis in the kidney of mice deficient in IL-17RA signaling. UUO was performed in WT and Il17ra^−/−^ mice (*n* = 7–12). At day 7 p.i., (a) the frozen kidney sections were assessed for collagen I and collagen III deposition by immunofluorescence staining. (b) UUO and non-UUO kidneys were evaluated for mRNA expression of Col1a1 and Col3a1 by qPCR. (c) Frequency of *α*SMA^+^ cells was evaluated by immunofluorescence staining. Photomicrographs are representative of 2 independent experiments for (a) and (c). Original magnification: 10x for (a) and (c). (d) Kidneys were evaluated for *α*SMA mRNA expression by qPCR. Single-cell suspension from the perfused kidneys was subjected to flow cytometry analysis to determine the percentages of kidney infiltrating (e) neutrophils and (f) macrophages (gated on CD45^+^ cells). In the scattered plots, each dot represents individual mouse and error bars indicate mean ± SD. The data are pooled from three independent experiments for (a–d) and (g) and two independent experiments for (e) and (f). *p* value ≤0.05 (^∗^), ≤0.01 (^∗∗^), ≤0.001 (^∗∗∗^), and ≤0.0001 (^∗∗∗∗^). ns: not significant.

Previous studies have shown that the balance between ECM proteins' synthesis and degradation determines the severity of ECM deposition and eventual development of TF [7]. To assess whether IL-17RA signaling negatively regulates the deposition of ECM proteins, we evaluated collagen I and collagen III deposition in the WT and *Il17ra^−/−^* kidney in mice with UUO. Indeed, immunofluorescence staining revealed increased deposition of collagen I and collagen III in the renal parenchyma of *Il17ra^−/−^* kidney following ureteral obstruction (Figure 4(a)).

We further determined whether exaggerated deposition of ECM proteins in the *Il17ra*^−/−^ mice could be due to increased expression of ECM protein genes. We measured transcript levels of *Col1a1* and *Col3a1* in the WT and *Il17ra^−/−^* kidneys following ureteral obstruction. UUO significantly enhanced transcript expression of *Col1a1* and *Col3a1* compared to non-UUO kidney. However, mRNA levels of *Col1a1* and *Col3a1* were comparable between the obstructed kidneys of WT and *Il17ra^−/−^* mice at day 7 post-surgery (Figure 4(b)). Additionally, immunofluorescence staining and qPCR revealed similar number of ECM protein secreting *α*-smooth muscle actin (*α*SMA^+^) myofibroblasts and *αSMA* mRNA expression between the groups, respectively (Figures 4(c) and 4(d)).

Following ureteral obstruction, macrophages infiltrate the kidney and facilitate TF by secreting profibrotic growth factors including TGF*β* [7]. A recent study has demonstrated a critical role of hematopoietic cell-specific IL-17R signaling in regulating TF [31]. To assess the impact of IL-17RA signaling on infiltrating macrophages, infiltration of inflammatory cells was evaluated in the obstructed kidneys of WT and *Il17ra^−/−^* mice. Seven days after surgery, significantly increased percentages of neutrophils (CD45^+^CD11b^+^Ly6G^+^) and macrophages (CD45^+^CD11b^+^F4/80^+^) were seen in the WT and *Il17ra^−/−^* obstructed kidneys (Figures 4(e) and 4(f)). While mice deficient in IL-17RA signaling showed diminished neutrophil infiltration, there was no difference in the frequency of macrophages (out of total kidney-infiltrating CD45^+^ cells) between the groups following UUO.

**Figure 5:**
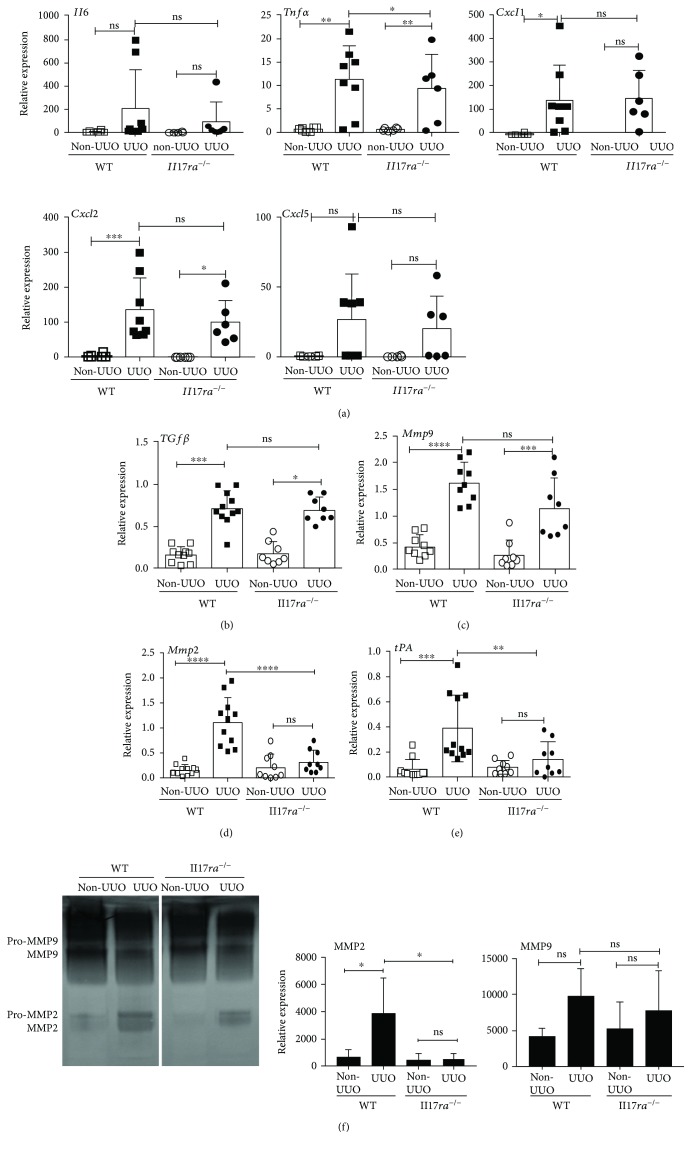
MMP2 and tPA expressions are compromised in the obstructed kidney of Il17ra^−/−^ mice. WT and Il17ra^−/−^ mice (*n* = 6–11) were subjected to UUO. At day 7 p.i., obstructed and non-UUO kidneys were evaluated for mRNA expression of (a) Il6, Tnf*α*, Cxcl1, Cxcl2, and Cxcl5, (b) Tgf*β*, (c) Mmp9, (d) Mmp2, and (e) tPA by qPCR. (f) Total kidney extracts were subjected to gelatin zymography to assess for MMP9 and MMP2 activities at day 7 post-UUO. The lanes were run on the same gel but were noncontiguous (white line). In the scattered plots, each dot represents individual mouse and error bars indicate mean ± SD. The data are pooled from three independent experiments. *p* value ≤0.01 (^∗∗^), ≤0.001 (^∗∗∗^), and ≤0.0001 (^∗∗∗∗^). ns: not significant.

When evaluated for IL-17-responsive inflammatory cytokines and chemokine gene expression, *Il17ra*^−/−^ kidney exhibited a significant reduction in *Tnfα* mRNA expression than control animals following UUO (Figure 5(a)). However, *Il6*, *Cxcl1*, *Cxcl2*, and *Cxcl5* transcript levels were comparable between the groups. Additionally, renal transcript expression of *Tgfβ* were comparable between the *Il17ra^−/−^* and WT UUO kidneys (Figure 5(b)). Collectively, these results suggest that exaggerated ECM protein deposition cannot be attributed to increased gene expression of ECM proteins, number of myofibroblasts, macrophage infiltration, and *Tgfβ* expression in the *Il17ra^−/−^* mice following UUO.

### 3.4. Renal Gene Expression of *Mmp2* and *tPA* Are Diminished in the Absence of IL-17R Signaling

Multiple studies have emphasized the essential role of ECM protein-degrading enzymes in the removal of fibrous tissue in various organs [32]. Thus, we sought to determine whether increased TF in *Il17ra*^−/−^ mice is due to diminished degradation of ECM proteins in the kidney. We measured the expression of *Mmp2*, *Mmp9*, and *tPA* genes in the obstructed kidney of WT and *Il17ra*^−/−^ mice. These genes are selected based on their known role in the development of organ fibrosis [32]. While *Mmp9* transcript expression was similar between the groups, renal mRNA levels of *Mmp2* and *tPA* were significantly reduced in the UUO kidneys of *Il17ra^−/−^* mice than WT animals (Figures 5(c)–5(e)). Accordingly, gelatin zymography of total kidney extracts revealed significantly reduced MMP2 but not MMP9 activity in the UUO kidney of *Il17ra^−/−^* mice than WT at day 7 p.i. (Figure 5(f)). Overall, this data suggests that diminished expression of MMP2 and tPA may result in the reduced degradation of ECM proteins and drive aberrant TF in *Il17ra^−/−^* mice.

### 3.5. Activation of IL-17-KKS-Axis Protects against TF by Inducing the Expression of Matrix-Degrading Enzymes

**Figure 6:**
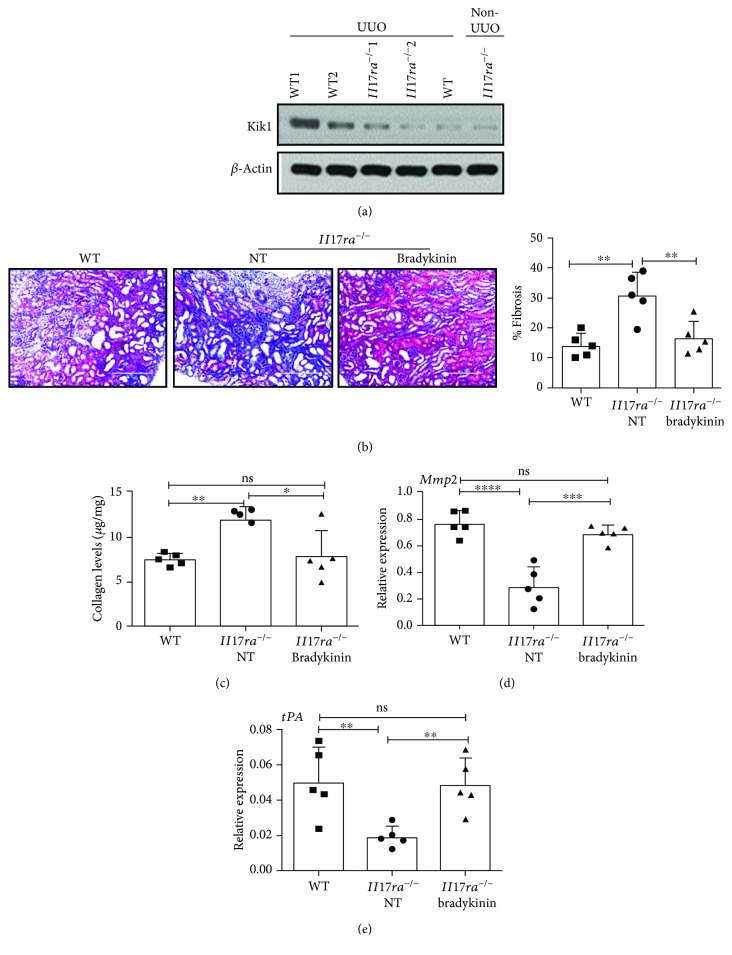
Diminished activation of KKS in the obstructed kidney of Il17ra^−/−^ mice. Surgically unilateral ureteral obstruction was performed in WT and Il17ra^−/−^ mice (*n* = 5). At day 7 post-surgery, (a) total kidney homogenate was assessed for Klk1 expression by immunoblot analyses. *β*-Actin is used as loading control. Representative immunoblot image from two independent experiments. (b) Il17ra^−/−^ mice (*n* = 5) were either treated with bradykinin (300 nmol/kg/day) or left untreated (PBS injected) starting on day −1 (relative to surgery) and then daily for the next 8 days. On day 0, mice were subjected to UUO and evaluated for TF at day 7 post-surgery. WT mice (*n* = 5) were subject to UUO and left untreated. The TF development was evaluated by Masson's trichome staining of UUO kidney sections. Photomicrographs are representative of 2 independent experiments. Original magnification: 20x. (c) The total collagen content in the kidney was measured by hydroxyproline assay. The renal transcript expression of (d) Mmp2 and (e) tPA was assessed by qPCR. In the scattered plots, each dot represents individual mouse and error bars indicate mean ± SD. The data are pooled from two independent experiments for (a–e). *p* value ≤0.05 (^∗^), ≤0.01 (^∗∗^), ≤0.001 (^∗∗∗^), and ≤0.0001 (^∗∗∗∗^). ns: not significant.

Based on the known antifibrotic functions of KKS activation and its implication in IL-17-mediated renal immunity, we next interrogated the role of Klk1 in IL-17-driven protection against TF [11]. At day 7 post-surgery, immunoblot analysis of kidney whole-cell extract revealed a strong suppression of Klk1 protein expression in the *Il17ra*^−/−^ mice compared to WT animals (Figure 6(a)). Collectively, this result confirms our prior findings and indicates that KKS is activated in the obstructed kidney in an IL-17-dependent manner.

Kallikreins cleave “kininogens” to generate kinins, namely, bradykinin and kallidin [18]. Numerous studies have identified an essential role of bradykinin in protection against TF following UUO [25, 26]. In this setting, bradykinin induced the renal expression of matrix-degrading enzymes such as Mmp2 and tPA, with minimal impact on ECM synthesis and macrophage influx in the UUO kidney [25, 26]. To define the contribution of IL-17-KKS-axis driven renal protection against TF, *Il17ra*^−/−^ mice were either treated with bradykinin or left untreated starting 3 days prior to UUO and then daily for the next 7 days. *Il17ra*^−/−^ mice treated with bradykinin showed reduced TF as evidenced by diminished fibrotic changes and total kidney collagen content compared to untreated *Il17ra*^−/−^ mice (Figures 6(b) and 6(c)). The level of total collagen content in the bradykinin treated kidney was comparable to untreated WT mice. Interestingly, the protective phenotype observed in *Il17ra^−/−^* mice receiving bradykinin correlated with increased renal expression of *Mmp2* and *tPA* genes following UUO (Figures 6(d) and 6(e)).

**Figure 7:**
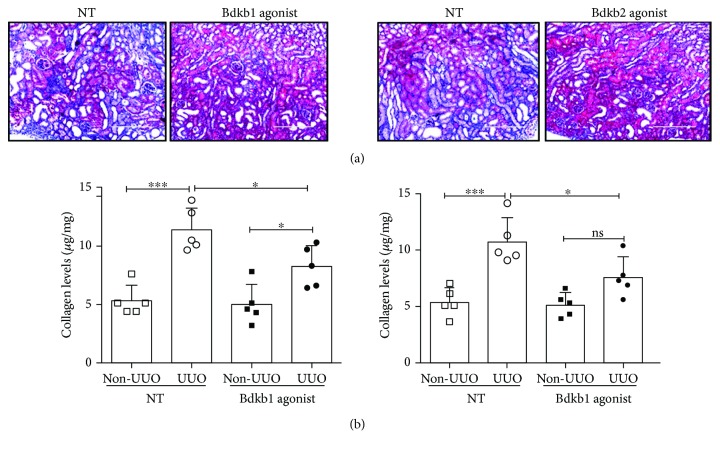
Activation of Bdkrb2 and Bdkrb1 which are key downstream mediators of IL-17-KKS axis-driven renal protection against TF. Groups of WT mice (*n* = 5) were either treated with selective agonist for Bdkrb1 or Bdkrb2 or left untreated (NT). Mice were evaluated for fibrosis development over a period of 7 days post-UUO surgery by (a) Masson's trichome staining and (b) measuring total collagen content in the UUO and non-UUO kidneys. Photomicrographs are representative of 2 independent experiments. Original magnification: 20x. In the scattered plots, each dot represents individual mouse and error bars indicate mean ± SD. The data are pooled from two independent experiments. *p* value ≤0.05 (^∗^) and ≤0.001 (^∗∗∗^). ns: not significant.

Bradykinin and its metabolite des-Ard-bradykinin activate Bdkrb2 and Bdkrb1, respectively. Based on our finding that the IL-17/KKS axis confers protection against TF, we next wanted to test the preclinical efficacy of Bdkrb agonists in treating mice with renal fibrosis. Accordingly, UUO was induced in WT mice and they were either treated with Bdkrb1 agonist or Bdkrb2 agonist or left untreated. At day 7 post-surgery, mice were evaluated for TF development by Masson's trichome staining of kidney sections and measuring the total collagen content in the obstructed kidney. As shown in Figures 7(a) and 7(b), we observed that treatment with either Bdkrb1 or Bdkrb2 agonists confers similar protection against TF. These data indicate that the IL-17-KKS axis facilitates the degradation of ECM protein in a Mmp2- and tPA-dependent manner.

## 4. Discussion

IL-17 promotes tissue inflammation and autoimmunity but also plays an important role in host defense against pathogens [33]. Consistent with its proinflammatory function, several studies have implicated IL-17 as a profibrotic cytokine. For example, IL-17 drives skin and pulmonary fibrosis, dilated cardiomyopathy, atherosclerosis, and hepatic fibrosis in experimental models. However, nagging discrepancies argue against these interpretations and experimental evidence also suggests an antifibrotic role of IL-17 in lung and skin fibrosis [34–38]. Compared to lung, skin, liver, and heart, studies investigating the role of IL-17 in renal fibrosis are surprisingly scarce. The few studies that have been performed have yielded contradictory results [14–16, 31]. Despite obvious parallels between fibrosis in the kidney and other organs, there are also a number of important differences in kidney and kidney-specific consequences. Renal specific factors, including poor regenerative capacity, toxins (uraemia), hypoxia, and arterial blood pressure significantly contribute to kidney disease outcomes [4]. Thus, lessons from skin, lung, or liver fibrosis cannot necessarily be applied to kidney. Understanding organ-specific differences has obvious therapeutic implications, since targeting kidney-specific factors would likely spare other organs from unwanted side effects. Here, we show that IL-17 is rapidly upregulated and plays an unappreciated antifibrotic role in the kidney following UUO. We have shown that IL-17R activation *in vivo* reduced UUO-induced TF and overexpression of IL-17 is beneficial in preventing renal fibrosis. IL-17 activates KKS, which in turn induces the expression of matrix-degrading enzymes in the obstructed kidney. These observations are consistent with the antifibrotic effect of KKS and particularly bradykinin in TF development.

In the past decade, major emphasis in the field of IL-17 has been placed on understanding how IL-17-producing cells are generated. Fewer resources have been dedicated on defining downstream signaling via IL-17R on target cells [33, 39]. The specific IL-17R signaling pathways and downstream cell targets involved in mediating protection against TF are unknown. Although IL-17R is ubiquitously expressed, most studies to date indicate that the essential IL-17 target cells are nonhematopoietic [40, 41]. A recent study reported that IL-17 may affect renal fibrosis by directly impacting macrophage development [31]. Here, we show that there is no contribution for direct IL-17R signaling in the recruitment of macrophages in the obstructed kidney. Rather, we show that IL-17 is produced locally in the obstructed kidney and renal cells can respond to IL-17 directly [11]. Future studies should take advantage of *Il17ra* conditional knockout mice to dissect downstream signaling events in kidney-resident cells in the pathogenesis of TF.

A published study suggests that IL-17 is important for driving TF in the mouse model of UUO. In this paper, the authors showed that IL-17 acts on T cells to produce RANTES, a chemokine required for inflammatory cell infiltration in the obstructed kidney [14]. Since RANTES can be induced by numerous cytokines, failure to show that *in vitro* stimulation of T cells with IL-17 drives RANTES production weakens the overall interpretation of this report. Additionally, T cells use preexisting mRNA to produce and secrete RANTES rapidly following TCR stimulation [42]. TF occurs in rapid response to a nonimmune stimulus. Thus, it is unlikely that in this short time frame kidney-migrating CD3^+^ T cells would encounter self-antigens to produce RANTES and drive disease pathogenesis in a UUO model of TF. In contrast, data from Sun et al. and our lab show that IL-17 plays an unappreciated kidney protective role in TF [15]. The apparent discordance between Peng et al. and our findings is currently unclear. However, we show that the seemingly opposite phenotype observed between these studies cannot be attributed to a difference in IL-17-driving gut microbiota between mice, as antibiotic treatment has minimal impact on TF development following UUO.

The accumulation of ECM proteins in pathological states results from an imbalance between both synthesis and degradation. We show here for the first time how the IL-17-KKS axis reduced ECM protein deposition by studying both ECM protein synthesis and degradation. In line with prior observations in bradykinin receptor-deficient mice, we observed no difference in collagen I and III protein synthesis between the obstructed kidneys of *Il17ra*^−/−^ and WT mice [25]. Rather, the alteration was noted in the degradation of ECM components due to diminished expression of matrix-degrading enzymes including Mmp2 and tPA. It has been reported that bradykinin is a potent stimulus for tPA and Mmp2 production in the kidney [25]. In addition, plasmin transforms metalloproteinases from their latent to active forms [43]. The paralleled decrease in tPA and Mmp2 production related to decreased ECM protein degradation, thus suggesting a role for the IL-17-Bradykinin/tPA/Mmp2 activity in protection against TF.

An increase in the aging population and incidence of diabetes and hypertension has contributed to an alarming rise in the prevalence of ESRD [4]. Antibodies against IL-17 and its receptor have been approved for the treatment of autoimmune diseases, but the potential side effects of blocking IL-17 are not well defined, particularly in the context of chronic kidney diseases [44]. While our data provide good evidence that IL-17 is needed for resistance against TF, the mechanisms that mediate this protection are poorly understood. Our new data implicate Klk1 providing an intriguing link between IL-17-mediated renal protection and activation of KKS. Treatment of TF patients with a combination of ACE inhibitor and angiotensin II receptor blockers shows limited efficacy and can be associated with persistent cough, angioedema, stenosis, birth defects, and renal failure [45]. Moreover, parenteral IL-17 as a possible therapy is likely to be associated with undesirable systemic inflammatory responses. Therefore, a comprehensive understanding of the inflammatory events in the kidney, and particularly the details of IL-17 signaling *in vivo*, is likely to be beneficial in designing new therapeutic or preventive approaches to treat TF.

Our discovery of an unrecognized connection between IL-17 and Klk1 suggests a previously unanticipated avenue for treatment of TF and is a major advance in our understanding of the function of IL-17 in the kidney. ACE inhibitors serve to increase levels of bradykinin and are routinely used to treat chronic kidney diseases [46]. Bdkrb2 agonist (lobradimil) has either been used or currently in clinical trials for treating brain tumors and HIV-infected individuals with cryptococcal meningitis (ClinicalTrials.gov: NCT00005602, NCT00019422, NCT00001502, and NCT00002316). Our data show a preclinical efficacy of treating TF with selective Bdkrb1 or Bdkrb2 agonists. Thus, exploiting IL-17-Klk1 pathways in preclinical immunotherapeutic modalities may dictate the development of new, safe, inexpensive, and rapidly implementable treatment options for TF with already available drugs.

## 5. Conclusions

- IL-17 plays an antifibrotic role in tubulointerstitial fibrosis following ureteral obstruction.
- IL-17 activates the kallikrein-kinin system and facilitates the degradation of ECM proteins via upregulation of matrix-degrading enzymes such as matrix-degrading enzyme-2 and tissue plasminogen activator.
- IL-17-kallikrein-kinin system axis-driven renal protection against TF is mediated by both bradykinin receptors 1 and 2.

## Data Availability

All the data used to support the findings of this study are included within the article. The raw data used to support the findings of this study are available from the corresponding author upon request.
